# Sodium-Ion Conductivity and Humidity-Sensing Properties of Na_2_O-MoO_3_-P_2_O_5_ Glass-Ceramics

**DOI:** 10.3390/nano12020240

**Published:** 2022-01-12

**Authors:** Mallaurie Foucaud, Sanja Renka, Teodoro Klaser, Jasminka Popović, Željko Skoko, Petr Mošner, Ladislav Koudelka, Ana Šantić

**Affiliations:** 1 Ruđer Bošković Institute, 10000 Zagreb, Croatia; mallaurie.foucaud@ecole.ensicaen.fr (M.F.); sanja.renka@irb.hr (S.R.); Teodoro.Klaser@irb.hr (T.K.); jpopovic@irb.hr (J.P.); 2 Department of Physics, Faculty of Science, University of Zagreb, 10000 Zagreb, Croatia; zskoko@phy.hr; 3 Department of General and Inorganic Chemistry, Faculty of Chemical Technology, University of Pardubice, 53210 Pardubice, Czech Republic; Petr.Mosner@upce.cz (P.M.); Ladislav.Koudelka@upce.cz (L.K.)

**Keywords:** sodium molybdenum phosphate glass-ceramics, crystallization, ionic conductivity, humidity sensors

## Abstract

A series of glass-ceramics were prepared by heat-treatments of 40Na_2_O-30MoO_3_-30P_2_O_5_ (in mol%) glass in a temperature range from 380 (*T*_g_) to 490 °C (*T*_c_) and for 1–24 h. The prepared glass-ceramics contain from 2 to 25 wt.% of crystalline NaMoO_2_PO_4_. The sodium-ion conductivity in these materials decreases up to one order of magnitude with an increase in the degree of crystallization due to the immobilization of sodium ions in crystalline NaMoO_2_PO_4_. The transport of sodium ions in these materials occurs primarily through the dominant continuous glassy phase, and it is weakly affected by the sporadically distributed crystalline grains. However, the prepared glass-ceramics exhibit high proton conductivity in a humid atmosphere and remarkable humidity-sensing properties; this could be related to crystalline NaMoO_2_PO_4_, which provides sites for water adsorption. The glass-ceramic prepared at 450 °C for 24 h shows the best humidity-sensing performance among all samples, showing an increase in proton conductivity for more than seven orders of magnitude with the increase in relative humidity from 0% to 95%. Under a highly humid atmosphere (95% relative humidity and 25 °C), the proton conductivity of this glass-ceramic reaches 5.2 × 10^−3^ (Ω cm)^−1^. Moreover, the electrical response of these materials on the change in the relative humidity is linear and reversible in the entire range of the relative humidity, which indicates that they are novel promising candidates for application as humidity sensors.

## 1. Introduction

The controlled crystallization of glasses is an advantageous procedure for the preparation of glass-ceramics with specific physicochemical properties [1,2]. According to the recently updated definition, glass-ceramics are “inorganic, non-metallic materials prepared by controlled crystallization of glasses which contain at least one type of functional crystalline phase and a residual glassy phase” [1]. In these materials, the volume fraction of the crystalline phase may vary from ppm to almost 100% [1]. Most commonly, controlled crystallization is achieved by proper heat-treatment of the glass, and by varying the heat-treatment conditions (mainly temperature and time), it is possible to tune the (micro)structure of the glass-ceramics, the amount of crystalline phase(s) and, in some cases, their composition. This, in turn, provides possibilities to increase mechanical toughness, tailor conductive pathways for ions and electrons and to form a template or framework for subsequent interaction with other materials [2]. All these valuable properties make glass-ceramics highly important for application in various areas, from medicine and dentistry, radioactive waste immobilization, optical and magnetic devices to energy storage and conversion technologies [2].

In the last several decades, a significant research effort has been directed toward the preparation and development of glass-ceramics with high electrical conductivity. Indeed, many studies have shown that ionic [3,4,5,6,7,8,9,10,11], as well as electronic (polaronic) [12,13,14,15,16,17,18,19,20], conductivity can be greatly enhanced by crystallization. In most of these studies, an increase in conductivity was found to be related to the interfacial regions between the nanocrystalline phase and the remnant glassy matrix, which provide easy conducting pathways for charge carriers, thus increasing the overall conductivity of the glass-ceramic. A detailed review on the effects of nanocrystallization on the transport properties of various glass-ceramic systems was recently published by Garbarczyk et al. [21]. From the applicative point of view, these findings offer a vast array of possibilities to implement highly conductive glass-ceramics in energy storage and conversion applications, such as electrolytes (ionically conducting glass-ceramics) and cathodes (electronically conducting glass-ceramics) in all-solid-state batteries in particular [11,18,21,22,23]. 

Recently, we studied a glass series with composition 40Na_2_O-*x*MoO_3_-(60-*x*)P_2_O_5,_ 0 ≤ *x* ≤ 50 mol% and showed that the replacement of the conventional glass former (P_2_O_5_) with the conditional glass-forming oxide (MoO_3_) causes a significant increase in sodium-ion conductivity with the maximum in conductivity at *x* = 30 mol% [24]. This effect, which is, by its nature, similar to the classical mixed glass former effect, is related to the formation of mixed phosphate–molybdate units in the glass network, which facilitates the mobility of sodium ions. 

In the present study, we investigated the influence of controlled crystallization on the ionic conductivity of the most conductive glass from this series, namely, 40Na_2_O-30MoO_3_-30P_2_O_5_ (in mol%) glass. The crystallization was induced by heat-treatments of glass at different temperatures and for different times in order to obtain glass-ceramics with different (micro)structures. Although crystallization resulted in a moderate decrease in sodium-ion conductivity with an increase in the amount of crystalline Na(MoO_2_)PO_4_, we show that prepared glass-ceramics exhibit very high sensitivity to humidity, which indicates their promising application as humidity sensors.

## 2. Materials and Methods

The parent glass 40Na_2_O-30MoO_3_-30P_2_O_5_ (in mol%) was prepared using the melt-quenching technique. The mixture of reagent grade chemicals Na_2_CO_3_, MoO_3_ and H_3_PO_4_ was calcinated by heating it up to 600 °C and by keeping it at that temperature for 2 h to remove water. The reaction mixture was then melted in a covered platinum crucible at 1000 °C for 15 min under the air atmosphere. The melt was subsequently poured into a preheated graphite mold, and the obtained glass was cooled to room temperature. The amorphous character of the prepared glass was checked by X-ray diffraction analysis.

Based on the glass transition temperature (*T*_g_) and glass crystallization temperature (*T*_c_), heat-treatment temperatures for induced crystallization were chosen to be 380, 450 and 490 °C. The powder of 40Na_2_O-30MoO_3_-30P_2_O_5_ glass was pressed in ~0.7-mm-thick pellets and heat-treated at these temperatures for 1, 12 and 24 h. The heat-treatments were performed in an Nabertherm LHT 04/17 furnace with a 10 °C/min heating rate and in a steady air atmosphere. The as-prepared glass-ceramics were labeled in accordance with their heat-treatment temperature and time; for instance, 450 C-12 h glass-ceramic was prepared by heating the glass at 450 °C for 12 h.

X-ray diffraction data of the prepared glass-ceramics were collected at RT on Bruker D8 Discover diffractometer (Bruker AXS GmbH, Karlsruhe, Germany) equipped with LYNXEYE XE-T detector, in Bragg–Brentano geometry. Rietveld structure refinement was performed in the HighScore Xpert Plus program 3.0 (Malvern Panalytical, Almelo, the Netherlands). Vesta (free crystallographic software available online: https://jp-minerals.org/vesta/en/download.html, ver. 3.5.7; accessed date: 4 November 2021) was used for crystal structure visualization [25]. Refinement was carried out by using the split-type pseudo-Voigt profile function and the polynomial background model. Isotropic vibration modes were assumed for all atoms. During the refinement, zero shift, scale factor, half-width parameters, asymmetry and peak shape parameters were simultaneously refined. Microstructural information was obtained in the course of Rietveld refinement with LaB_6_ used as instrumental broadening standard. The fractions of the amorphous/crystalline phases were determined using Rietveld refinement by the addition of crystalline Al_2_O_3_ as an internal standard. The content of the amorphous phase was determined using the internal standard method. In a system with a known amount of standard *S*, the weight content of the crystalline phase *p* is determined by the following formula:

$$W_{p}=\frac{W_{P}S_{p}{\left(ZMV\right)}_{p}}{S_{s}{\left(ZMV\right)}_{s}}\cdot \frac{1}{1-W_{s}}$$

while the amount of the amorphous phase is determined by the following expression:

*W_am_* = 1 − *W_s_* − ∑*W_p_*

where *W_s_*, *W_p_* and *W_am_* are weight percentages of the standard, crystalline phase and amorphous phase, respectively; *S* is the scale factor; *Z* is the number of the formula unit within the unit cell; *M* is formula unit mass; and *V* is the volume of the unit cell. 

The microstructure and elemental analysis of the glass-ceramics were studied by field-emission scanning electron microscopy FE-SEM JSM 7000 (JEOL, Welwyn Garden City, UK) equipped with the Oxford Instruments EDS/INCA 350 energy dispersive X-ray analyzer (EDS).

For the electrical measurements, gold electrodes (3.8 mm in diameter) were sputtered on both surfaces of the samples using Sputter Coater SC7620 (Quorum Technologies LTD, Newhaven, UK). The samples were subsequently placed between two brass electrodes, and the complex impedance was measured using an impedance analyzer (Novocontrol Alpha-AN Dielectric Spectrometer, Novocontrol Technologies GmbH & Co. KG, Montabaur, Germany) in a wide frequency (0.01–10^6^ Hz) range in dry nitrogen. The AC amplitude was 100 mV. The measurements of all samples were taken isothermally from −30 to 240 °C, with a temperature step of 30 °C. The temperature was controlled to an accuracy of ±0.2 °C. At each temperature, samples were thermally equilibrated for at least 20 min prior to the measurement. For humidity-sensing measurements, the complex impedance was measured in the same frequency range (0.01–10^6^ Hz) at room temperature (25 °C) and different relative humidity (RH) values: 49% (air, ambient conditions), 79%, 84% and 95% in a home-made sample cell for humidity measurements. The relative humidity inside the sample cell was obtained using saturated aqueous solutions of different salts: NaCl (RH = 79%), KCl (RH = 84%) and K_2_SO_4_ (RH = 95%). The impedance spectra at different relative humidity conditions were measured consecutively until the sample reached the constant impedance spectrum. After that, we continued to record the impedance spectra for 2 h to test the stability of the material response. The impedance spectra were analyzed by equivalent circuit modeling using the complex nonlinear least-squares fitting procedure (ZView software, ver. 2.70, Scribner Associates, SAD). From the values of electrical resistance (*R*) and electrode dimensions (*A* is the electrode area, and *d* is the sample thickness), the DC conductivity was calculated according to the following equation: *σ*_DC_ = *d*/(*A*·*R*).

## 3. Results and Discussion 

### 3.1. Crystallization and Structure of Glass-Ceramics

The temperatures of the heat-treatments of the 40Na_2_O-30MoO_3_-30P_2_O_5_ (mol%) glass were selected based on the reported DSC results [26]: 380 °C (glass transition temperature, *T*_g_), 450 °C (arbitrary temperature between *T*_g_ and *T*_c_) and 490 °C (crystallization temperature, *T*_c_). At each temperature, glass pellets were heated for 1, 12 and 24 h as described in the previous section. 

**Figure 1:**
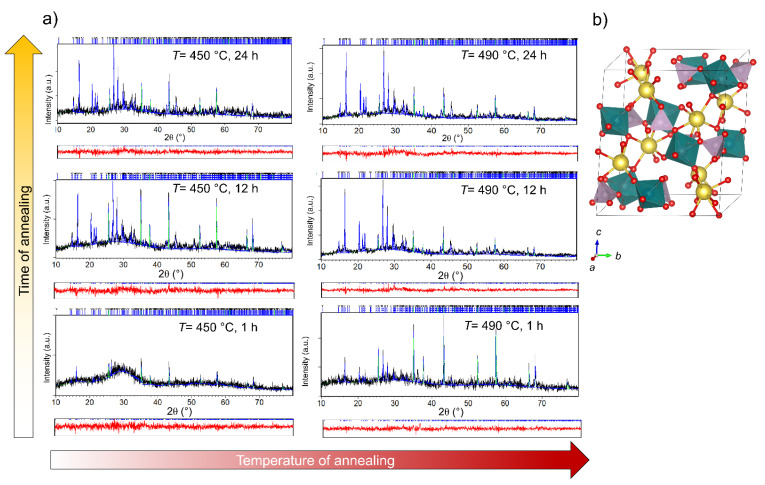
(**a**) Rietveld refinements of glass samples crystallized at 450 and 490 °C for 1, 12 and 24 h, mixed with powder of Al_2_O_3_ used as an internal standard for amorphous phase quantification. Experimental data are given by the black line, the calculated pattern is shown in blue, and the red line below represents the difference curve. Blue vertical marks show the positions of diffraction lines belonging to NaMoO_2_PO_4_ phase, while the positions of Al_2_O_3_ lines are given as green vertical marks. (**b**) The structure of NaMoO_2_PO_4_: Mo is given in teal, P in pink, Na in yellow and O in red.

The effect of temperature and time of annealing on the evolution of crystalline phases was investigated by X-ray powder diffraction (XRPD). The heat-treatment at 380 °C, irrespective of the heat-treatment time, does not induce crystallization processes. The XRPD pattern (not shown) of these samples exhibits a wide scattering halo without any diffraction lines, indicating a fully amorphous structure. However, the Rietveld refinement analysis performed for the glass pellets heat-treated at 450 and 490 °C for the time duration of 1, 12 and 24 h revealed the crystallization of sodium molybdate-phosphate NaMoO_2_PO_4_ (see Figure 1a).

The Rietveld analysis shows that heat-treatment at 450 °C for 1 h induces very weak crystallization, since the 450 C-1 h sample is characterized by short-range ordering with some traces (~2 wt.%) of NaMoO_2_PO_4_. With the increase in the heat-treatment time to 12 h, the amount of crystalline NaMoO_2_PO_4_ increases to 20 wt.%; however, with a further increase to 24 h, the crystallinity of the glass-ceramics remains the same, see Table 1. However, an increase in the degree of crystallization was observed with the increase in the temperature to 490 °C; the sample heat-treated at 490 °C for 1 h contains 11 wt.% of crystalline NaMoO_2_PO_4_ and 89 wt.% of the amorphous phase, while the amount of crystalline phase increases to ~25 wt.% for heat-treatments of 12 and 24 h. The quantitative composition of all samples is given in Table 1.

The formation of crystalline NaMoO_2_PO_4_ agrees well with previously published results on the crystallization of sodium molybdate phosphate glasses [26,27]. The NaMoO_2_PO_4_ phase crystallizes in the monoclinic system, *P* 2_1_/*n* with refined unit cell parameters: *a* = 6.3601(9) Å, *b* = 11.929(2) Å, *c* = 13.454(3) Å and *β* = 116.37(1) ° similar to those reported by Kierkegaard [28]. The structure of NaMoO_2_PO_4_ consists of distorted MoO_6_ octahedra, which are connected in a corner-shared manner to PO_4_ tetrahedra and NaO_7_ polyhedral groups, as shown in Figure 1b.

Based on the composition of the glass-ceramics given in Table 1, it can be observed that the crystallization of NaMoO_2_PO_4_ is promoted by an increase in heat-treatment temperature; however, at both temperatures, 450 and 490 °C, the crystallization process completes within 12 h. A relatively low maximal amount of crystalline phase of ~25 wt.% in the prepared glass-ceramics can be related to two factors: (i) the stoichiometry of the parent glass, which differs from the stoichiometry of NaMoO_2_PO_4_ (theoretical percent yield is ~66 wt.%), and (ii) the tendency of NaMoO_2_PO_4_ to nucleate and grow predominantly via the surface crystallization mechanism [27], thereby hindering voluminous crystallization.

The X-ray line-broadening analysis performed on the 450 C-12 h and 490 C-24 h samples revealed pronounced growth of the diffraction domain sizes of the NaMoO_2_PO_4_ from ~80 to ~120 nm with an increase in heat-treatment temperature.

**Figure 2:**
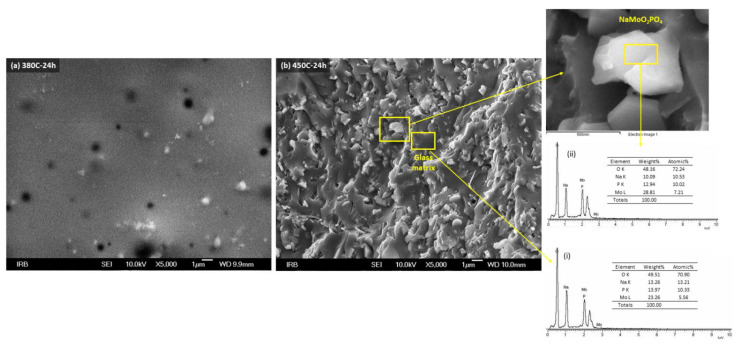
SEM micrographs of samples prepared at (**a**) 380 °C for 24 h and (**b**) 450 °C for 24 h, and EDS spectra from selected areas of 450 C-24 h sample.

An insight into the microstructural features of the glass-ceramics was gained using scanning electron microscopy. Samples prepared at 380 °C for all three heat-treatment times as well as those prepared at 450 °C for 1 h show homogeneous morphology with sporadic cracks and holes as can be seen in Figure 2a for the 380 C-24 h sample. This is in line with the XRD results, which confirmed a fully amorphous structure or traces of crystalline NaMoO_2_PO_4_ in these samples. The morphology of the glass-ceramics with a higher degree of crystallinity (450 C-12 h, 450 C-24 h, 490 C-1 h, 490 C-12 h and 490 C-24 h) contains small irregular grains randomly distributed in the glassy matrix as can be seen in Figure 2b for the glass-ceramics prepared by heating at 450 °C for 24 h. The EDS analysis showed that the chemical composition of the grains matches the NaMoO_2_PO_4_ phase, whereas the composition of the matrix corresponds to the glassy phase (see right-hand panel in Figure 2b). The size of NaMoO_2_PO_4_ grains is approximately ~1 μm; however, the X-ray line-broadening analysis revealed that the crystallites within the grains have an average size of ~80 nm.

### 3.2. Sodium-Ion Conductivity 

**Figure 3:**
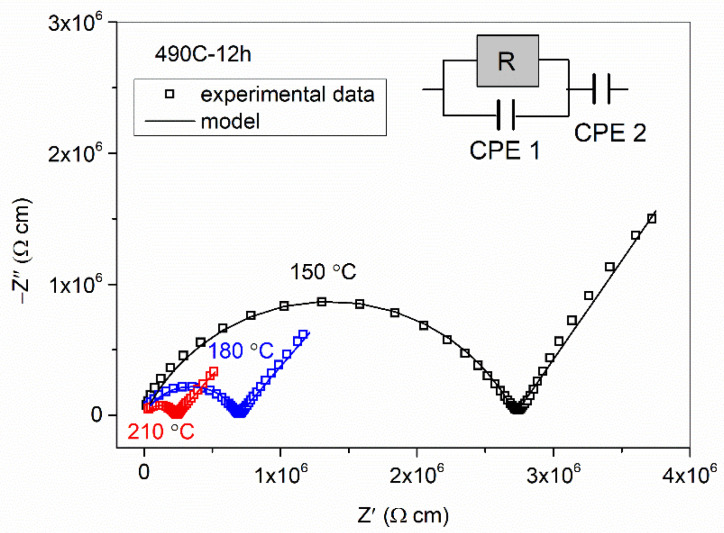
Nyquist plot of glass-ceramic prepared at 490 °C for 12 h measured at 150, 180 and 210 °C in dry nitrogen.

The Nyquist plot for the 490 C-12 h glass-ceramic, as a typical plot for all samples in this study, is shown in Figure 3. At each temperature, the plot consists of a single semicircle and a low-frequency spur. While the impedance semicircle corresponds to the conduction through the bulk, the spur is related to the electrode polarization effect, i.e., the blocking of sodium ions on the surface of the metallic electrode, and it becomes more pronounced as the temperature increases due to an increase in the mobility of the ions. Here, it should be noted that none of the glass-ceramics show additional spectral features, such as a second semicircle, the presence of which is usually related to the blocking effects of the secondary phase on the transport of ions. This result suggests that the transport of sodium ions occurs through a continuous amorphous glass matrix and is weakly affected by the crystalline NaMoO_2_PO_4_. This is in line with the XRPD and SEM results, which show randomly distributed isolated grains of NaMoO_2_PO_4_ embedded in the dominant amorphous glassy phase.

The corresponding equivalent electrical circuit for the impedance plots shown in Figure 3 consists of a parallel combination of a resistor and constant phase element (CPE 1), which models the bulk response, and an additional constant phase element (CPE 2) in the series, which models the electrode polarization. The CPE is an empirical impedance function of the type Z*_CPE_ = 1/[*A*(i*ω*)*^α^*], where *ω* = 2π*f*, *f* is the measuring frequency, i = (−1)^1/2^ is the imaginary unit, *A* is the constant, and *α* is a power law exponent with a value in the range of 0 < *α* < 1 [29]. For *α* = 1, the CPE acts as an ideal capacitor, whereas for *α* = 0, it is a resistor. The fitting parameters (resistance *R* and parameters *A* and *α*) at various temperatures for the 490 C-12 h glass-ceramic are given in Table 2. At the lowest temperatures, −30, 0 and 30 °C, the Nyquist plot shows a single semicircle without a low-frequency spur since, at these temperatures, sodium ions are weakly mobile and do not produce electrode polarization. With a further increase in temperature, the mobility of the ions increases, and electrode polarization appears and becomes more and more pronounced. The value of the parameter *A*_1_, representing a pseudo-capacitance, ranges from 10^−12^ to 10^−10^ s^α^ Ω^−1^, whereas parameter *A*_2_ exhibits significantly larger values, 10^−7^–10^−5^ s^α^ Ω^−1^, which is in agreement with the capacitance of the bulk and the sample–electrode interface, respectively [30].

**Figure 4:**
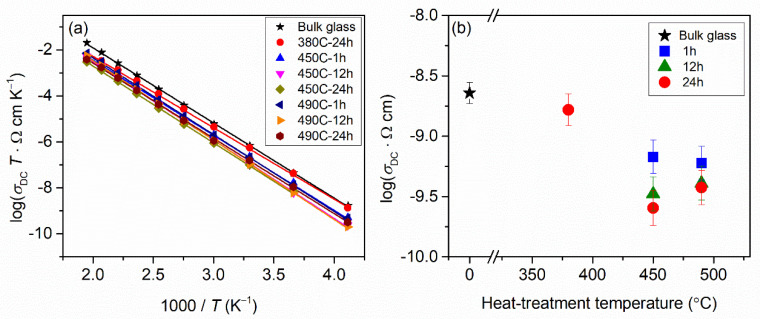
(**a**) Arrhenius plot for glass and glass-ceramics and (**b**) their DC conductivity, *σ*_DC_, at 30 °C as a function of heat-treatment temperature and time. The error bars in (**a**) are at most of the order of the symbol size.

From the values of resistance (*R*) and electrode geometry, DC conductivity was calculated as explained in the Materials and Methods Section. For all samples, the DC conductivity exhibits Arrhenius temperature dependence (see Figure 4a) and, hence, has characteristic activation energy. The activation energy for DC conductivity, *E*_DC_, for each sample was determined from the slope of log(*σ*_DC_*T*) vs. 1000/*T* using the equation *σ*_DC_*T* = *σ*_0_exp(−*E*_DC_/k_B_*T*), where *σ*_DC_ is the conductivity, *σ*_0_ is the pre-exponential factor, k_B_ is the Boltzmann constant, and *T* is the temperature (K). The DC conductivity at 30 °C and the determined activation energy, *E*_DC_, for the parent glass and prepared glass-ceramics are shown in Table 3. The activation energies, *E*_DC_, for the glass-ceramics are nearly constant and very close to the activation energy for the parent glass, which supports our conclusion that the conduction process in all glass-ceramics takes place primarily through the amorphous glass matrix. 

Figure 4b shows the DC conductivity of the glass-ceramics as a function of the heat-treatment conditions.

As can be seen from Figure 4b, the sample heat-treated at 380 °C for 24 h shows only a slightly smaller *σ*_DC_ than that of the parent glass, which is consistent with its fully amorphous structure. With an increase in the heat-treatment time and temperature, the DC conductivity of the glass-ceramics decreases for up to one order of magnitude compared to the parent glass. At both heat-treatment temperatures, an increase in the heat-treatment time causes a decrease in conductivity, which is directly related to the formation of crystalline NaMoO_2_PO_4_. As previously noted, the charge carriers in these glass-ceramics are sodium ions in the glassy phase, so an increase in the fraction of crystalline NaMoO_2_PO_4_, in which sodium ions are immobilized, implies a decrease in the overall concentration of the mobile sodium ions and, hence, a decrease in their conductivity. Further, these results suggest that the interfacial regions between the crystalline grains and amorphous matrix in these materials do not provide easy pathways for ionic conductivity as observed in some other glass-ceramic systems [3,4,5,6]. One possible reason could be that these regions are not interconnected since there is a relatively small fraction of sporadically distributed isolated crystalline grains in these materials, so their contribution to the conduction process is negligible.

Moreover, it is worth noting that the glass-ceramics prepared at 450 °C for 12 and 24 h exhibit slightly lower conductivity than those prepared at 490 °C for the same heat-treatment times despite having a lower fraction of crystalline phase ~20 wt.% vs. ~25 wt.%. This somewhat reversed trend is related to the effect of porosity. Upon preparation, the 450 C-24 h sample showed a more porous structure than 490 C-24 h glass-ceramic, which implies lesser connectivity between amorphous/crystalline grains and, consequently, hindered ionic transport.

Overall, the above results reveal that the sodium-ion conductivity in glass-ceramics moderately decreases with the formation of crystalline NaMoO_2_PO_4_ grains. This rather disappointing result seriously limits the application of these materials from the point of view of sodium-ion conductivity. However, in the following section, we show that the presence of crystalline NaMoO_2_PO_4_ grains opens up a completely different applicative area for these glass-ceramics—that of humidity sensors.

### 3.3. Humidity-Sensing Properties

**Figure 5:**
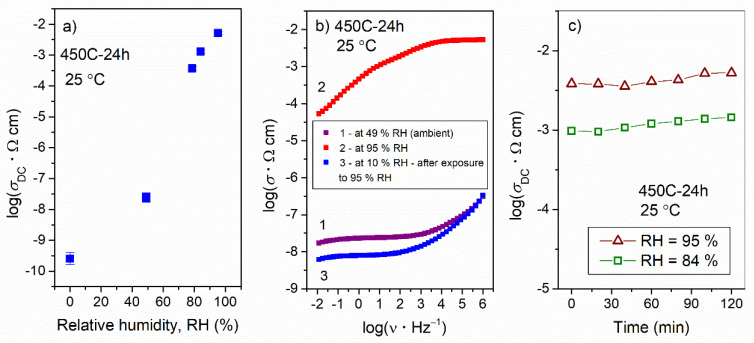
(**a**) DC conductivity at room temperature as a function of relative humidity (RH), (**b**) recovery experiment: 1st measurement—conductivity spectrum at RH = 49% (ambient), 2nd measurement—conductivity spectrum at RH = 95% for 2 h and 3rd measurement—conductivity spectrum at RH ≈ 10% recorded 12 h after exposure to high humidity and (**c**) time-dependent conductivity at RH = 84% and 95% for glass-ceramics prepared at 450 °C for 24 h. Note that the strong electrode polarization effect in the conductivity spectrum measured at RH = 95% (Figure b) is due to proton conductivity, i.e., blocking of protons at the surface of the electrode.

In the course of the electrical characterization of the glass-ceramics prepared at 450 and 490 °C, it was found that their electrical conductivity differed significantly depending on whether the measurements were performed in ambient air or dry nitrogen. While conductivity measured in dry nitrogen was of the order of ~10^−10^ (Ω cm)^−1^ (see Table 3 and the discussion in the previous section), it increased for several orders of magnitude in the room conditions. This fortuitous finding motivated us to investigate the influence of different relative humidity conditions on the electrical properties of the prepared samples. Figure 5a displays the changes in DC conductivity with changes in relative humidity from RH = 0% to RH = 95% for the glass-ceramic prepared at 450 °C for 24 h. As can be seen from the figure, the DC conductivity at room temperature increased nearly linearly for more than seven orders of magnitude with an increase in relative humidity, reaching a value of *σ*_DC_ = 5.2 × 10^−3^ (Ω cm)^−1^ at RH = 95%. This huge increase in conductivity is related to the transport of protons due to adsorbed water on the surface of the glass-ceramic.

It is known that, in porous ceramics, the mechanism of water-related conduction includes the physical adsorption of water molecules at the grain surface and water condensation in the pores depending on the relative humidity [31,32]. At low humidity, water molecules are chemically adsorbed at preferential sites at the grain surface (chemisorbed layer), onto which subsequent layers of water molecules are physically adsorbed (physisorbed layer). The physisorbed water dissociates due to high electrostatic fields in the chemisorbed layer, 2H_2_O ⟷ H_3_O^+^ + OH^−^, and charge transport occurs when H_3_O^+^ releases a proton to a neighboring water molecule, which accepts it while releasing another proton, and so forth (Grotthuss chain reaction). However, at high humidity, liquid water condenses in the pores and electrolytic conduction takes place along with the proton transport in the adsorbed layers [31].

In our study, all of the glass-ceramics prepared at 450 and 490 °C exhibited humidity-sensing behavior; this arises from the presence of crystalline NaMoO_2_PO_4_, which provides sites for the adsorption of water (note that the fully amorphous samples prepared by heat-treatment at 380 °C do not exhibit humidity-dependent conductivity) and porous microstructure. In line with this, we observed that the 450 C-24 h sample showed the best humidity-sensing performance in terms of the change in magnitude of conductivity with the change in relative humidity (see Figure 5a). Such a high sensitivity to humidity is related to the combination of two parameters: (i) relatively high crystallinity (19 wt.%) and (ii) high porosity (higher than that of the glass-ceramics prepared at 490 °C). Here, it should be noted that the response/recovery time of the 450 C-24 h glass-ceramic is rather slow; the response time which is defined as the time required for 90% of the total change in conductivity is 10 min for humidification from dry nitrogen to 49% RH, whereas the recovery time (desiccation from 84% RH to dry nitrogen) is 80 min. Such a slow response/recovery time represents a limitation for the application of this material as a fast-responding humidity sensor; however, different (micro)structural characteristics (porosity and fraction of crystalline phase) might result in improved operational performance. Moreover, it is important to note that no change in the structure of the 450 C-24 h glass-ceramic was observed after exposure to a highly humid atmosphere, which indicates the chemical stability of the material. This is also reflected in the reversible electrical response to the changes in humidity conditions shown in Figure 5b. As can be seen from the figure, the conductivity of the 450 C-24 h glass-ceramic after exposure to RH = 95% for 2 h reverts to the value that is very close to the one measured before the humidity-dependent measurements. Moreover, the time-dependent impedance measurements reveal that the 450 C-24 h glass-ceramic maintains stable conductivity for a period of at least 2 h (maximum duration of the measurement), see Figure 5c, which is very beneficial for practical applications of this material.

Finally, it is worth noting that the 450 C-24 h glass-ceramic exhibits a significantly higher rate of change in conductivity as a function of the relative humidity than other humidity-sensitive glass-ceramics available in the literature, for example, those based on La_2_O_3_-TiO_2_-V_2_O_5_ [33] lithium titanium phosphate [34] and barium titanate, lithium niobate and zinc orthosilicate containing silver particles [35]. Therefore, our results show the high potential of Na_2_O-MoO_3_-P_2_O_5_ glass-ceramics for application as humidity sensors. However, since humidity-sensing performance depends on various factors, it is of further interest to investigate the humidity-sensing properties of these glass-ceramics, in particular, their behavior in a wider range of fractions of crystalline NaMoO_2_PO_4_ and different porosity. These studies are under way.

## 4. Conclusions

A series of glass-ceramics were prepared by heat-treatments of 40Na_2_O-30MoO_3_-30P_2_O_5_ (in mol%) glass at 380 (*T*_g_), 450 and 490 °C (*T*_c_) and for various times (1, 12 and 24 h). The XRPD analysis shows that the heat-treatment at 380 °C, irrespective of the heat-treatment time, does not induce crystallization processes, whereas at higher temperatures, NaMoO_2_PO_4_ crystallizes. With the increase in the heat-treatment temperature from 450 to 490 °C, the amount of crystalline phase increases and reaches a maximal value of 25 wt.%; however, at both temperatures, the crystallization process completes within 12 h. The sodium-ion conductivity decreases for up to one order of magnitude with the increase in the fraction of crystalline NaMoO_2_PO_4_, which is related to the immobilization of the fraction of sodium ions in the crystals. The prepared glass-ceramics show excellent humidity-sensing properties due to NaMoO_2_PO_4_ crystals, which provide sites for the adsorption of water. The proton conductivity of the best-performing glass-ceramic prepared at 450 °C and for 24 h increases by more than seven orders of magnitude with the increase in relative humidity from 0% to 95%. Based on the high proton conductivity, as well as the linear and reversible electrical response over the entire relative humidity range, these glass-ceramics appear to be excellent novel candidates for application as humidity sensors.

## Figures and Tables

**Table 1:** Quantitative composition of the samples heat-treated for 1–24 h at 380, 450 and 490 °C.

Sample	Heat-Treatment Conditions		Composition (in wt.%)		R (Weighted Profile) (%)	Diffraction Domain Size (nm)
	*T* (°C)	*t* (h)	NaMoO_2_PO_4_	Amorphous		
380 C-1-24 h	380	1, 12, 24	-	100	-	-
450 C-1 h	450	1	2(1)	98(1)	9.31	85(8)
450 C-12 h	450	12	20(1)	80(1)	8.87	98(8)
450 C-24 h	450	24	19(1)	81(1)	8.56	117(10)
490 C-1 h	490	1	11(2)	89(2)	9.46	81(8)
490 C-12 h	490	12	25(1)	75(1)	7.99	103(9)
490 C-24 h	490	24	24(1)	76(1)	7.38	119(10)

**Table 2:** The fitting parameters of impedance spectra at various temperatures for the glass-ceramic prepared at 490 °C for 12 h.

Sample 490 C-12 h	Equivalent Circuit Parameters				
Temperature	*R* (Ω)	CPE 1		CPE 2	
		*A*_1_ (s^α^ Ω^−1^)	*α*_1_	*A*_2_ (s^α^ Ω^−1^)	*α*_2_
−30 °C	1.14 × 10^12^	3.61 × 10^−12^	0.76	-	-
0 °C	3.78 × 10^10^	8.52 × 10^−12^	0.74	-	-
30 °C	1.65 × 10^9^	2.21 × 10^−11^	0.74	-	-
60 °C	2.17 × 10^8^	3.87 × 10^−11^	0.74	2.78 × 10^−7^	0.34
90 °C	3.27 × 10^7^	6.58 × 10^−11^	0.73	1.14 × 10^−6^	0.39
120 °C	6.28 × 10^6^	1.03 × 10^−10^	0.73	3.50 × 10^−6^	0.47
150 °C	2.12 × 10^6^	1.52 × 10^−10^	0.72	3.89 × 10^−6^	0.63
180 °C	5.39 × 10^5^	1.91 × 10^−10^	0.72	7.42 × 10^−6^	0.56
210 °C	1.85 × 10^5^	2.65 × 10^−10^	0.71	1.35 × 10^−5^	0.55
240 °C	7.08 × 10^4^	3.09 × 10^−10^	0.72	1.79 × 10^−5^	0.53

**Table 3:** DC conductivity, *σ*_DC_, at 30 °C and activation energy, *E*_DC_, for parent 40Na_2_O-30MoO_3_-30P_2_O_5_ (in mol%) glass and prepared glass-ceramics.

Sample	*σ*_DC_ (Ω cm)^−1^ at 30 °C ± 1.0%	*E*_DC_ (eV) ± 1.0%
Glass *	2.28 × 10^−9^	0.65
380 C-24 h	1.65 × 10^−9^	0.62
450 C-1 h	6.72 × 10^−10^	0.65
450 C-12 h	3.32 × 10^−10^	0.68
450 C-24 h	2.54 × 10^−10^	0.66
490 C-1 h	5.97 × 10^−10^	0.66
490 C-12 h	4.08 × 10^−10^	0.69
490 C-24 h	3.75 × 10^−10^	0.65

* Data taken from [24].

## Data Availability

Not applicable.
